# Mapping the Binding Interface of PET Tracer Molecules and Alzheimer Disease Aβ Fibrils by Using MAS Solid‐State NMR Spectroscopy

**DOI:** 10.1002/cbic.202000143

**Published:** 2020-05-19

**Authors:** Zheng Niu, Riddhiman Sarkar, Michaela Aichler, Hans‐Jürgen Wester, Behrooz Hooshyar Yousefi, Bernd Reif

**Affiliations:** ^1^ Munich Center for Integrated Protein Science (CIPS−M) Department Chemie Technische Universität München Lichtenbergstrasse 4 85747 Garching Germany; ^2^ Helmholtz-Zentrum München Institute of Structural Biology (STB) Ingolstädter Landstrasse 1 85764 Neuherberg Germany; ^3^ Helmholtz Zentrum München Research Unit Analytical Pathology (AAP) Ingolstädter Landstrasse 1 85764 Neuherberg Germany; ^4^ Technische Universität München Department of Pharmaceutical Radiochemistry Walther-Meißner-Strasse 3 85748 Garching Germany; ^5^ Philipps University of Marburg Department of Nuclear Medicine Baldingerstrasse. 1 35043 Marburg Germany

**Keywords:** Alzheimer's disease, amyloid-beta fibrils, deuteration, imaging tracer, magic angle spinning solid-state NMR spectroscopy, positron emission tomography

## Abstract

Positron emission tomography (PET) tracer molecules like thioflavin T specifically recognize amyloid deposition in brain tissue by selective binding to hydrophobic or aromatic surface grooves on the β‐sheet surface along the fibril axis. The molecular basis of this interaction is, however, not well understood. We have employed magic angle spinning (MAS) solid‐state NMR spectroscopy to characterize Aβ‐PET tracer complexes at atomic resolution. We established a titration protocol by using bovine serum albumin as a carrier to transfer hydrophobic small molecules to Aβ(1‐40) fibrillar aggregates. The same Aβ(1‐40) amyloid fibril sample was employed in subsequent titrations to minimize systematic errors that potentially arise from sample preparation. In the experiments, the small molecules ^13^C‐methylated Pittsburgh compound B (PiB) as well as a novel Aβ tracer based on a diarylbithiazole (DABTA) scaffold were employed. Classical ^13^C‐detected as well as proton‐detected spectra of protonated and perdeuterated samples with back‐substituted protons, respectively, were acquired and analyzed. After titration of the tracers, chemical‐shift perturbations were observed in the loop region involving residues Gly25‐Lys28 and Ile32‐Gly33, thus suggesting that the PET tracer molecules interact with the loop region connecting β‐sheets β1 and β2 in Aβ fibrils. We found that titration of the PiB derivatives suppressed fibril polymorphism and stabilized the amyloid fibril structure.

## Graphical abstract

**Binding PET tracer molecules to amyloid fibrils**: PET tracers specifically recognize amyloid deposition in neurodegeneration, although the molecular basis of this interaction is not well understood. MAS solid‐state NMR spectroscopy is used to characterize Aβ‐PET tracer complexes. A bovine serum albumin protocol enables the titration of the hydrophobic molecules.

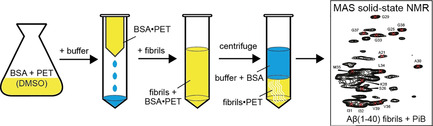

## Introduction

One of the pathological hallmarks of Alzheimer's disease (AD) is the deposition of amyloid plaques. These plaques are composed primarily of amyloid‐β peptides (Aβ), which is a cleavage product after processing of the amyloid precursor protein (APP) that consists of 36–43 amino acids.^1^ Positron emission tomography (PET) is employed to visualize amyloid deposits *in vivo,* enabling a non‐invasive diagnosis of AD. Several PET tracers were derived initially from thioflavin‐T (ThT), a small molecule that binds unspecifically to all classes of amyloids.^2^ It is thought that ThT specifically recognizes amyloid deposits in brain tissue by binding to hydrophobic or aromatic surface grooves on the β‐sheet surface along the fibril axis.^3^ The earliest amyloid imaging probes used within a clinical setup were [^18^F]FDDNP (2‐(1‐{6‐[(2‐[^18^F]fluoroethyl)(methyl)amino]‐2‐naphthyl}ethylidene) malononitrile)^4^ and *N*‐methyl‐[^11^C]2‐(4′‐methylaminophenyl)‐6‐hydroxybenzothiazole, termed “Pittsburgh compound B” (PiB).^5^ All reported radiotracers interact unspecifically with low specificity and selectivity to all amyloid aggregates. Differentiation among the different neurodegenerative diseases (e. g., Parkinson's disease and Alzheimer's disease) has so far not been possible.^6^ Recently, molecules have been developed which specifically bind to Aβ and not to α‐synuclein or tau fibrils and potentially enable an early diagnosis of a particular neurodegenerative disease.^7^ A particularly promising series of compounds is based on the molecule 4,4′‐diaryl‐2,2′‐bithiazole (DABTA). The selected DABTA molecules display a high affinity for Aβ42 (*K*_i_≃4 nM) and selectivity (>160‐fold) for Aβ as opposed to other proteinopathies by introduction of adequate functional groups.^8^ Preliminary *in vivo* data suggest a high brain uptake and fast clearance, encouraging the implementation of DABTA‐derived molecules as PET tracers with good pharmacokinetics.

MAS solid‐state NMR spectroscopy yields atom‐specific information on the structure of aggregates such as amyloid fibrils formed by the Alzheimer's disease peptides Aβ,^9^ α‐synuclein,^10^ or tau.^11^ NMR spectroscopy is particularly sensitive to changes in the local electronic environment and allows ligand binding to be probed. It was shown that the dye Congo Red interacts with the groove and orients itself along the fibril axis.^12^ However, the exact binding mechanism between PET tracer molecules and amyloid fibrils are so far not well understood. In the future, MAS solid‐state NMR can yield insights for the development of the next generation of PET tracers for AD diagnosis.

## Results

### Solid‐state NMR titration by BSA carrier system

**Figure 1:**
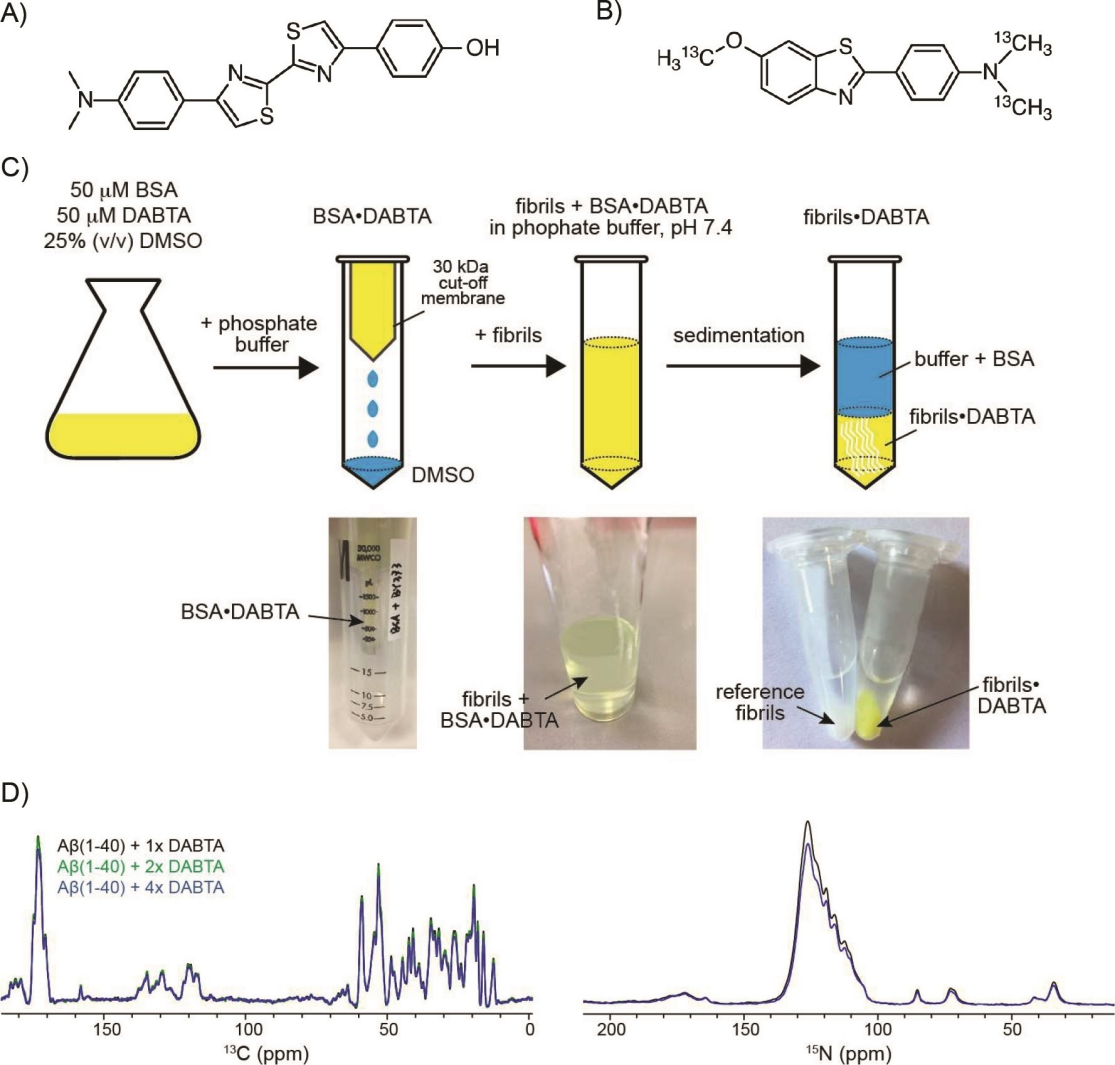
Chemical structures of A) 4,4′‐diaryl‐2,2′‐bithiazole (DABTA) and B) ^13^C‐methylated PiB (^13^C−Me−PiB). The carbon atoms of the dimethylamine as well as the methoxy groups of PiB were ^13^C enriched. C) Schematic representation of the titration protocol to transfer the PET tracer molecule to pre‐formed Aβ(1‐40) fibrils for MAS solid‐state NMR experiments. D) Superposition of 1D ^13^C and ^15^N spectra of Aβ(1‐40) fibrils titrated with the DABTA molecule at different molar ratios. The spectra were acquired on a Bruker Avance III 750 MHz spectrometer at 3 °C, setting the MAS rotation frequency to 10 kHz. Subsequent spectra with increasing DABTA concentration were recorded by unpacking and re‐suspending the sample in a buffer containing an increasing concentration of BSA‐bound DABTA.

PET tracer molecules (Figure 1A,B) are very hydrophobic and poorly soluble in aqueous buffer (<1 nM). To avoid organic solvents that potentially interfere with amyloid fibril integrity and to overcome the solubility issue, we suggest to use bovine serum albumin (BSA) as a carrier system to titrate hydrophobic molecules to pre‐formed amyloid aggregates (Figure 1C). Albumin is the most abundant serum protein that serves as a transport vehicle for several endogenous compounds including fatty acids, hemin, bilirubin and tryptophan, which bind albumin with high affinity.^13^ Due to its promiscuous binding properties, it has been suggested as a tool for drug delivery and as vehicle for clinical, biophysical and industrial purposes.^14^

In the following, the titration of DABTA to Aβ(1‐40) fibrils is described. DABTA has a characteristic yellow color in solution, which allows the individual titration steps to be followed by visual inspection. Using a UV meter, the titration of the PiB derivative can be followed in a similar way. First, DABTA is dissolved in a buffer containing 25 % (*v*/*v*) DMSO, together with an equimolar amount of BSA. Under these conditions, the DABTA molecules are sufficiently soluble. At the same time, BSA is still folded and able to bind to the hydrophobic molecule. In a subsequent step, the BSA•DABTA complex is washed with phosphate buffer to eliminate all DMSO. Finally, this solution is titrated to pre‐formed Aβ(1‐40) fibrils. To prepare a MAS solid‐state NMR sample, the fibril ⋅ ligand complex is pelleted by centrifugation, and packed into the MAS rotor.

After titration, a clear color change of the Aβ(1‐40) fibrils from white to yellow was observed, indicating that DABTA molecules bound to Aβ(1‐40) fibrils. Conversely, the supernatant did not contain any DABTA molecules after sedimentation. After the first round of NMR experiments, this procedure was repeated with increasing concentrations of BSA‐bound DABTA. For this purpose, the sample was unpacked from the solid‐state NMR rotor and washed with the BSA⋅DABTA buffer again. In order to prepare a sample of DABTA bound to Aβ(1‐40) fibrils at a 1 : 1 stoichiometry, an equimolar amount of fibrillar Aβ(1‐40) and BSA⋅DABTA was mixed together. The UV/Vis signal of the supernatant after sedimentation of the fibril⋅DABTA aggregates indicated that DABTA binds stoichiometrically to Aβ(1‐40) fibrils. The NMR signal intensity and the resolution of the Aβ(1‐40) fibril sample in complex with a onefold, twofold and fourfold molar excess of DABTA were comparable to the spectrum obtained for the Aβ(1‐40) fibril reference sample (Figure 1D), suggesting that the titration protocol with BSA as a carrier system works successfully without a significant loss of material for the individual titration steps.

### Structural characterization of the interaction between the ligands and Aβ(1‐40) fibrils

**Figure 2:**
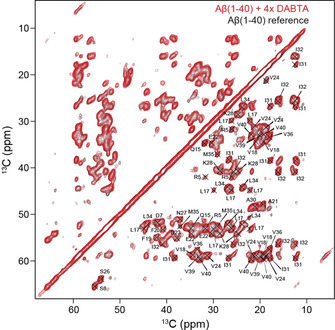
Superposition of 2D ^13^C,^13^C PDSD spectra of ^13^C,^15^N‐labeled Aβ(1‐40) fibrils (black) and Aβ(1‐40) fibrils with a fourfold molar excess of DABTA (red), recorded on a Bruker Avance III 750 MHz spectrometer. The MAS rotation frequency was adjusted to 10 kHz MAS. The ^13^C,^13^C spin diffusion mixing time was set to 50 ms.

We first carried out MAS solid‐state NMR experiments employing a protonated ^13^C,^15^N‐labeled Aβ(1‐40) fibril sample, and recorded ^13^C‐detected experiments to identify the binding site of the DABTA molecule. Aβ(1‐40) fibrils were grown by seeding as described in the Experimental Section. Fibril polymorphism was observed previously in Aβ fibril preparations.^15^ To ensure that the spectra obtained from the reference fibril and the fibril ⋅ DABTA sample are not different because of different fibril morphologies, we recorded experiments first using the reference sample, un‐packed this sample from the rotor after the first round of experiments and titrated then DABTA to the fibrils using the BSA protocol described above. Figure 2 and Figure S1 in the Supporting Information show 2D ^13^C,^13^C and 2D ^15^N,^13^C correlation solid‐state NMR spectra in presence and absence of DABTA. We observe a single set of resonances for all aliphatic carbons with ^13^C linewidths on the order of 120–165 Hz, suggesting that the pre‐formed Aβ(1‐40) fibrils have a high degree of homogeneity. Resonances were assigned using 3D NCACX and NCOCX type experiments. The assigned residues are summarized in Figure S2. We find that chemical shift changes between the DABTA bound fibrils and the reference fibril sample are very small and cannot be quantified reliably.

**Figure 3:**
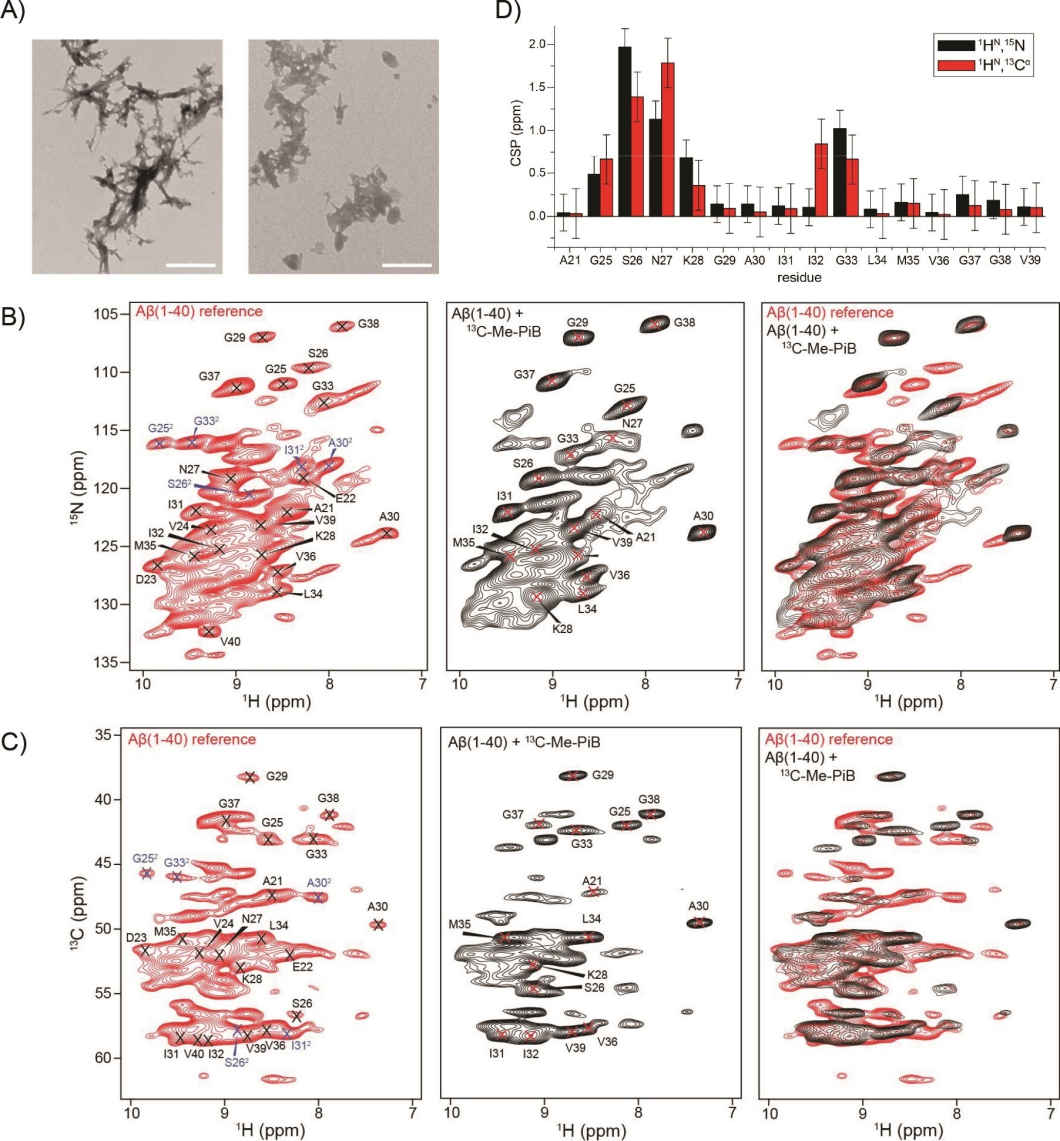
A) TEM images of Aβ(1‐40) fibrils (left), and Aβ(1‐40) fibrils in the presence of a fourfold molar excess of DABTA (right); scale bars: 500 nm. Individual and superimposed B) 2D ^1^H,^15^N correlation spectra and C) 2D ^1^H^N^,^13^C^α^ correlation spectra of ^2^H,^13^C,^15^N‐labeled Aβ(1‐40) fibrils in the absence (red) and presence (black) of an equimolar amount of ^13^C−Me−PiB. For the reference fibril sample, two sets of resonances are observed for the loop residues G25, S26, as well as for A30, I31 and G33 (indicated in blue). These peaks are strongly attenuated in the presence of ^13^C−Me−PiB. Fibrils were formed in phosphate buffer (prepared with H_2_O) to yield full protonation at exchangeable sites. 2D ^1^H^N^,^15^N and ^1^H^N^,^13^C^α^ correlation spectra were recorded on a Bruker Avance III 800 MHz spectrometer. The MAS rotation frequency was adjusted to 52 kHz. The effective sample temperature was kept constant at around 15 °C. D) Chemical shift perturbations (CSP) for ^1^H^N^,^15^N and ^1^H^N^,^13^C^α^ resonances obtained for a fibril reference sample and a fibril sample incubated with an equimolar amount of ^13^C−Me−PiB. CSPs were calculated from Equations 1 and 2 in the Experimental Section.

The similarity of the spectra suggests that DABTA has no dramatic impact on the amyloid fibril structure. This is also confirmed by a TEM analysis which indicates that the morphology of the DABTA⋅Aβ fibril co‐aggregates is very similar to the morphology of the Aβ(1‐40) reference fibrils (Figure 3A). Very similar results are obtained in case PiB was titrated to protonated Aβ(1‐40) fibrils (Figure S3). Surprisingly, the experimental CSPs are in both cases smaller compared to the shift changes observed for fibrils in the presence and absence of sulindac sulfide.^16^ The BSA titration experiments clearly indicate that the PET tracer molecules and Aβ fibrils interact (Figure 1C): In presence of the DABTA, the pelleted fibrils show a yellow color, whereas no DABTA can be detected in the supernatant. Apparently, the sensitivity of ^13^C for subtle changes in the chemical in environment are too small to be detected in regular ^13^C‐detected MAS solid‐state NMR experiments. We therefore prepared a perdeuterated, ^13^C,^15^N‐labeled Aβ(1‐40) fibril sample and isotopically enriched ^13^C−Me−PiB in order to better understand the interaction mechanism. Aβ(1‐40) fibrils were again grown as described previously.9a ^13^C−Me−PiB can be easily isotopically labeled by methylation of the free amine and hydroxy groups in dry acetone using ^13^C‐methyl triflate and ^13^C‐methyl iodide at 80 °C for 30–120 min (Figure 1B). At the same time, we envisioned that ligand‐based NMR experiments can be used to monitor interactions from the perspective of the ligand. To prepare fibrils, perdeuterated Aβ(1‐40) was incubated in protonated phosphate buffer to yield full protonation at exchangeable sites. Subsequently, ^13^C−Me−PiB was titrated to the deuterated Aβ(1‐40) fibrils as described above. The respective 2D ^1^H,^15^N correlation spectrum is shown in Figure 3B, yielding a very similar chemical shift pattern as observed previously.^17^ Backbone amide chemical shift assignments were obtained using hCANH and hcoCAcoNH.^18^ The assigned residues are summarized in Figure S4. ^1^H^N^,^13^C^α^ correlation spectra recorded using long‐range Hartmann‐Hahn mixing times^19^ are shown in Figure 3C. In contrast to the ^13^C‐detected experiments using protonated samples, clear chemical shift changes e. g. for residues Gly25, Ser26, Asn27 are observed. Figure 3D summarizes the experimental ^1^H^N^,^15^N and ^1^H^N^,^13^C^α^ chemical shift changes as a function of residue for Aβ(1‐40) titrated with an equimolar amount of ^13^C−Me−PiB. The largest shift changes are found for the loop region connecting the two β‐strands (involving residues Gly25‐Lys28), and for residues Ile32 and Gly33 that are located in the strand β2.

**Figure 4:**
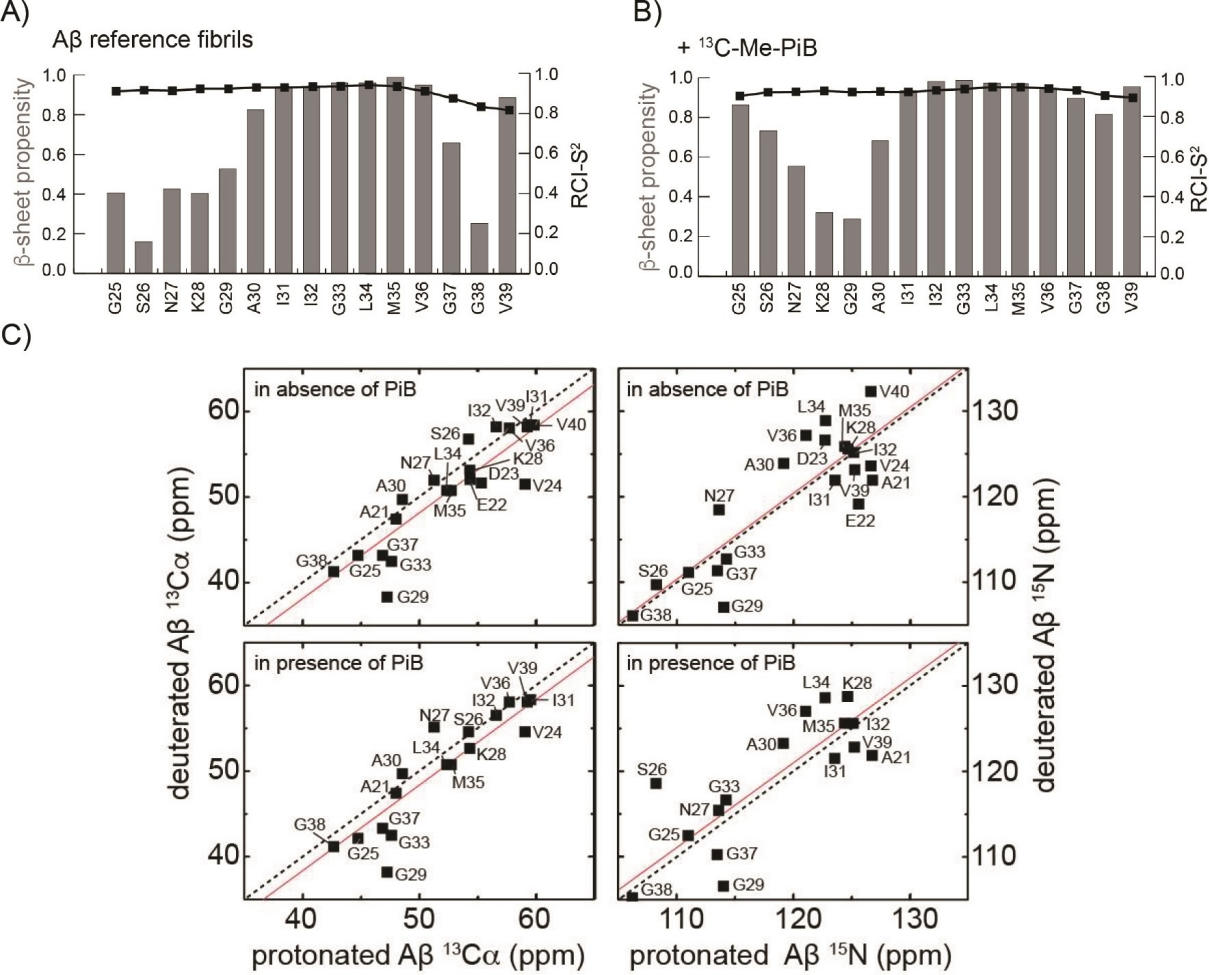
Random‐coil index squared order parameter values RCI−S^2^ (black squares)^20^ and secondary structure prediction using TALOS+ (gray rectangles)^21^ for A) Aβ(1‐40) reference fibrils and B) Aβ(1‐40) fibrils in the presence of ^13^C−Me−PiB. RCI−S^2^ scales are between 0 (total disorder) and 1 (fully rigid). After titration of PiB, in particular the C terminus of Aβ(1‐40) fibrils appears to adopt an increased β‐sheet propensity. C) Correlation of ^13^C^α^ and ^15^N chemical shifts of protonated and deuterated Aβ(1‐40) fibrils with (bottom) and without (top) ^13^C−Me−PiB. The black dashed and the red line indicate the diagonal and the linear fit, respectively. The ^13^C^α^ chemical shifts of the deuterated fibril sample are −1.809 ppm and −1.659 ppm, smaller in comparison than the values obtained for the protonated sample due to an isotope‐induced chemical shift change.^22^

We have suggested previously that hydrophobic cavities in the amyloid fibril structure are potential ligand binding sites.16a The hydrophobic cavities involving residues Gln15‐Leu17 /Val36−Val40 or Phe19−Ala21/Ile32‐Leu34 are potentially able to provide enough space for binding, whereas the hydrophilic loop region seems not suitable to accommodate the ligand. Interestingly, we observe two sets of resonances for the loop residues Gly25, Ser26, as well as for Ala30, Ile31 and Gly33 in the Aβ(1‐40) reference fibril spectra (Figure 3). This is reminiscent of spectra that were obtained previously for Aβ fibrils.9a There, however, all residues are affected which let us conclude that the basic building block in the amyloid fibril structure is an asymmetric dimer. After titration of ^13^C−Me−PiB to the Aβ(1‐40) fibril sample, this second set of crosspeaks is strongly decreased in intensity, suggesting that ^13^C−Me−PiB is reducing structural heterogeneity of the Aβ(1‐40) fibril. We assume that the PET tracer molecules induce a subtle structural difference in the loop region to provide space for binding of the ligand, and at the same time increase the stability of the amyloid fibril structure. On the basis of the chemical shift assignments, we calculated the random‐coil index squared order parameters (RCI−S^2^)^20^ and the secondary structure propensity using TALOS+^21^ (Figure 4). Residues Val18−Ala21 and Ala30−Val40 adopt β‐strand conformation (β1 and β2), whereas residues Glu22−Gly29 form a loop structure that connects the two β‐strands through side chain/side chain interactions. We find that the β‐sheet propensity is increased in presence of PiB. Chemical shift changes appear to be more pronounced for the perdeuterated sample, in comparison to chemical shift perturbations observed for the protonated sample. In order to find out whether protonated and deuterated fibril samples are structurally similar, we correlated ^13^C^α^ and ^15^N chemical shifts. Most assigned residues yield a linear correlation (Figure 4C), suggesting that deuterated and the protonated Aβ(1‐40) fibrils are comparable. The ^13^C^α^ chemical shifts in the absence and presence of PiB show an offset of −1.81 and −1.66 ppm which is due to an isotope‐induced chemical shift change as described in previously.^22^ We explain the increased ability of the proton to detect changes of the chemical environment by the fact that protons are closer to a potential ligand binding site. At the same time, protons have the largest gyromagnetic ratio, turning them into the most sensitive nucleus to detect changes of the local electronic environment. In addition, carbon resonances are broadened due to evolution of the homonuclear carbon, carbon scalar couplings which induce line widths on the order of 150–200 Hz in the ^13^C dimension. By contrast, proton resonances are not affected as heteronuclear interactions are easily removed by application of a decoupling scheme.

**Figure 5:**
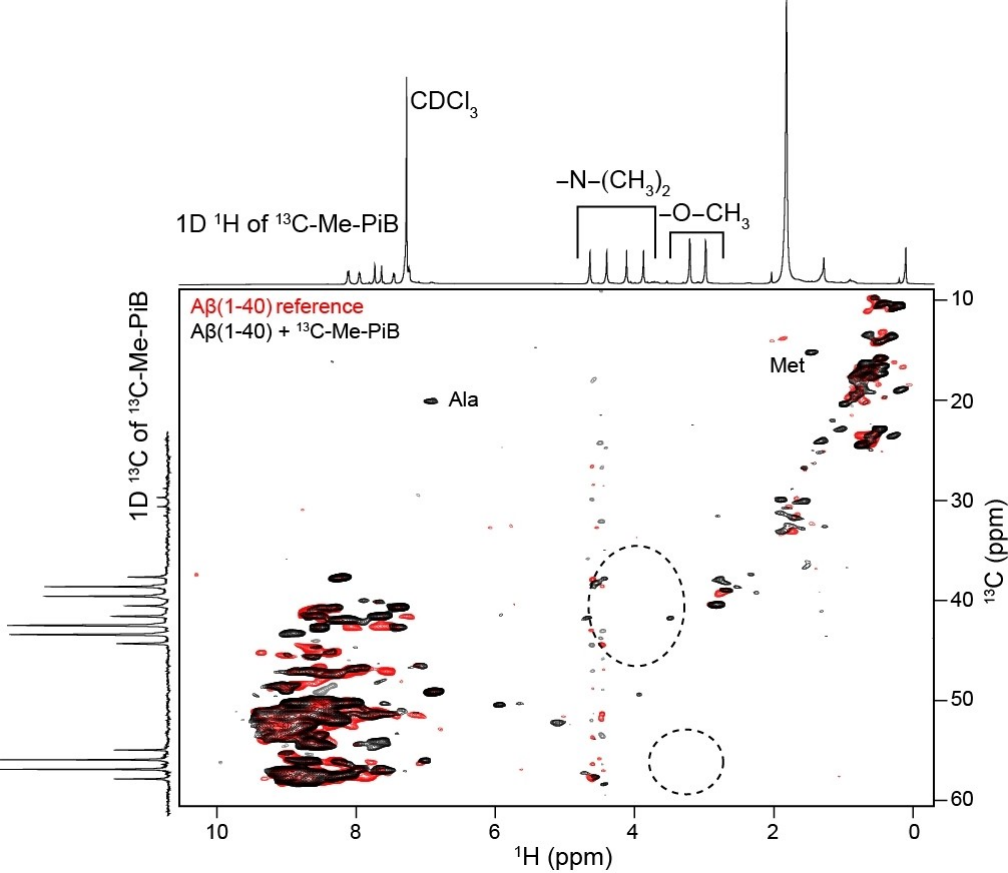
Superposition of 2D ^1^H,^13^C correlation spectra of ^2^H,^13^C,^15^N‐labeled Aβ(1‐40) fibrils in the absence (red) and presence (black) of an equimolar amount of ^13^C−Me−PiB. Fibrils were grown in a protonated phosphate buffer to yield full protonation at exchangeable sites. Spectra represented along the ^1^H and ^13^C chemical shift axes show the 1D ^1^H and ^13^C spectra of ^13^C−Me−PiB dissolved in deuterated chloroform. The dashed circles indicate the spectral regions for methoxy and *N*‐Me groups in ^13^C−Me−PiB. The 2D ^1^H,^13^C correlation spectra were recorded on a Bruker Avance III 800 MHz spectrometer. The MAS rotation frequency was adjusted to 52 kHz. The effective sample temperature was kept constant around 15 °C.

To identify the ligand resonances in the NMR spectra, we carried out 2D ^1^H,^13^C correlation experiments (Figure 5). We observe methyl ^1^H,^13^C correlation peaks for Aβ(1‐40) even though the peptide was perdeuterated. These resonances are due to protons at natural abundance as the carbon source employed for bacterial growth is not 100 % enriched with deuterons.^23^ The proton signals of ^13^C−Me−PiB are not detectable in a straightforward manner. The region of the spectrum in which ligand resonances are supposed to occur is indicated with two dashed circles. We speculate that the ligand is not visible in the NMR spectra, since we were employing a MAS rotation frequency of only 52 kHz. It turned out that this frequency is not high enough in case the proton density around the N‐methyl and methoxy groups of the fully protonated ^13^C−Me−PiB is too high.^24^ Alternatively, chemical exchange among different binding sites might be the reason for the small chemical shift changes observed in the ^13^C‐detected experiments. In fact, several distinct binding sites have been identified for the ThT class of Alzheimer's disease PET imaging agents for β‐amyloid fibrils.^25^ Even though the affinity for an individual binding site is in the nanomolar regime, multiple binding sites complicate the NMR spectroscopic analysis. A larger excess of the ligand is in this case required, which in turn hampers the exact characterization of the binding pocket, since chemical shift changes cannot be assigned any longer to a single binding event. In addition, faster MAS experiments are necessary to overcome methyl line broadening for this kind of samples.

## Conclusions

We have demonstrated that BSA is a useful carrier system that allows very hydrophobic molecules such as PET tracer molecules to be titrated to an amyloid fibril without the use of organic solvents such as DMSO for NMR experiments. In addition, we have shown that binding of ^13^C−Me−PiB induces chemical shift changes for residues Gly25−Lys28 and Ile32−Gly33 in Aβ(1‐40) fibrils, thus suggesting that ^13^C−Me−PiB binds to the hydrophobic binding pocket involving residues Ala21/Ile32 close to the turn in the Aβ fibril structure. In presence of the small molecule, peak doubling is not any longer observable. This suggests that ^13^C−Me−PiB stabilizes a particular Aβ fibril polymorph and reduces conformational dynamics.

## Experimental Section

**Expression, purification and fibrils preparation**: Aβ(1‐40) was expressed and purified as described previously.^26^ The M9 medium was supplemented with ^13^C‐gluocose and ^15^NH_4_Cl. Similarly, triply labeled Aβ(1‐40) peptide was produced. Instead of protonated water and glucose, D_2_O and D‐glucose‐^13^C_6_,1,2,3,4,5,6,6‐D_7_ was employed. Cell pellets were resuspended in 20 mM Tris ⋅ HCl buffer (pH 8.0). Subsequently, cell pellets were sonicated and dissolved with lysis buffer (8 M guanidinium chloride, 20 mM Tris ⋅ HCl pH 8.0). The dissolved inclusion bodies were centrifuged for 30 min at 20 000 rpm *using a* fiberlite F21‐8×50y fixed‐angle rotor (ThermoFisher).

The supernatant was filtered using a syringe filter equipped with a 0.22 μm MWCO membrane, and loaded onto a reverse phase chromatography SOURCE 30 RPC column. The HPLC column was first equilibrated using 80 % buffer A (10 mM ammonia solution) and 20 % buffer B (80 % acetonitrile and 0.3 % trifluoroacetic acid). The concentration of buffer B was then increased using a linear gradient (20 to 60 %) of buffer B. The Aβ(1‐40) peptide eluted at a concentration of 40–45 % of buffer B. The eluted Aβ(1‐40) peptide was lyophilized and stored in the freezer at −80 °C.

To prepare Aβ fibrils, the Aβ(1‐40) peptide powder was dissolved in 10 mM NaOH. The solution was centrifuged at 13 000 rpm (using a Thermo Scientific™ Sorvall^TM^ Legend^TM^ Micro 21R microcentrifuge equipped with a 24×1.5/2.0 mL standard rotor) for 10 min to remove any potential aggregates, and subsequently diluted into 50 mM phosphate buffer (pH 7.4; supplemented with 50 mM NaCl, 0.1 % NaN_3_), yielding a final Aβ(1‐40) peptide concentration of 50 μM. From now on, Aβ(1‐40) fibrils were grown in phosphate buffer under physiological condition. In order to obtain a homogeneous fibril sample, fibril growth was seeded over 12 generations.9a The first fibril generation was prepared by employing 100 μg of Aβ (in a volume of 0.5 mL). Fibrils were grown under continuous agitation (120 rotations/min) for 4 days. Fibril formation was regularly monitored by TEM. The second generation of fibrils was prepared by addition of seeds (10 % *w*/*w*) from generation 1 to a fresh Aβ solution (using again 100 μg of Aβ in a volume of 0.5 mL). The same concentration was employed in all subsequent generations. Prior to seeding, fibrils were fragmented by sonification for 10 min. Generation 2 was grown then for 2 days under agitation. The same protocol was employed for all subsequent generations, with the exception that fibrils were grown only for 2 h for generations 3–6, and 4 h for generations 7–12. Finally, in order to produce the solid‐state NMR Aβ fibril sample, 10 mg uniformly ^13^C,^15^N enriched Aβ peptide was monomerized employing the protocol described above. Fibril formation was initiated by seeding (10 % *w*/*w*) the solution with pre‐formed sonicated fibrils. Fibrils were grown at 37 °C with agitation for two weeks. The growth and the quality of Aβ fibrils was monitored by TEM by taking an aliquot from the Aβ40 fibril preparation.

**Solid‐state NMR experiments**: Two‐ and three‐dimensional solid‐state NMR spectra of uniformly ^13^C,^15^N‐labeled Aβ(1‐40) fibrils were recorded on a Bruker Avance III 750 MHz spectrometer, equipped with a triple‐resonance (^1^H,^13^C,^15^N) 3.2 mm MAS probe. In all ^13^C‐detected experiments, the MAS rotation frequency was adjusted to 10 kHz. High‐power proton decoupling (*ω*_RF_=71 kHz) was applied during acquisition using SPINAL‐64. For ^1^H,^13^C magnetization transfer, cross polarization was employed. ^13^C,^13^C transfers were achieved via PDSD using a mixing time of 50 ms. For assignment, 3D NCA/NCO, NCACX and NCOCX experiments were employed.^27^ Selective transfer of polarization between ^15^N and ^13^C was achieved using SPECIFIC CP.^28^ All spectra were processed using TopSpin 3.5 and CCPNmr 2.3.^29^

In all ^1^H‐detected experiments, the MAS rotation frequency was adjusted to ∼52 kHz. In the CP‐based HSQC experiment, the 90° pulses were set to 1.55 μs using a ^1^H RF field of 161 kHz. For ^15^N, a rf‐field amplitude of 50 kHz was employed corresponding to a 90° pulse length of 5.0 μs. ^1^H,^15^N CP was achieved using a contact time of 0.6 ms. WALTZ‐16 was applied for ^15^N decoupling during ^1^H acquisition. For 3D assignment experiments, ^13^C 90° pulses of 3.2 μs (*ω*_RF_=78 kHz), and ^15^N 90° pulses of 5.0 μs (*ω*_RF_=50 kHz) were employed. For the H−Cα/CO long range CP experiment, a contact time of 3.5 ms was used. There, the field on the ^13^C channel was locked using a RF field of 14 kHz. For both the CO−N and the Cα−N CP step, a contact time of 8 ms was employed, using a constant‐amplitude spin lock of about 33 kHz on ^13^C, and a tangent‐modulated amplitude spin lock of mean rf‐field amplitude of about 25 kHz on ^15^N. Data were acquired in the States‐TPPI mode in the indirect dimension. For the last magnetization transfer step in all pulse sequences an optimal control pulse sequence element was employed to yield maximum sensitivity.^30^ Backbone amide chemical shift assignments were obtained using hCANH and hcoCAcoNH triple resonance experiments.^18^

Chemical shift perturbations (CSPs) were calculated according to

(1) $$CSP\left({}^{1}H^{N},{}^{15}N\right)=\;\sqrt{{\left({{\delta H}^{N}}_{A\beta -PiB}-{{\delta H}^{N}}_{A\beta }\right)}^{2}+\frac{{\left({\delta N}_{A\beta -PiB}-{\delta N}_{A\beta }\right)}^{2}}{25}}$$

and

(2) $$CSP\left({}^{1}H^{N},{}^{13}C^{\alpha }\right)=\;\sqrt{{\left({{\delta H}^{N}}_{A\beta -PiB}-{{\delta H}^{N}}_{A\beta }\right)}^{2}+\frac{{\left({\delta C\alpha }_{A\beta -PiB}-{\delta C\alpha }_{A\beta }\right)}^{2}}{4}}$$

respectively.

Error bars have been estimated assuming average ^1^H^N^,^15^N and ^13^C^α^ line widths of 0.21, 0.75 and 0.5 ppm respectively.

## Conflict of interest

The authors declare no conflict of interest.

## Supporting information

As a service to our authors and readers, this journal provides supporting information supplied by the authors. Such materials are peer reviewed and may be re‐organized for online delivery, but are not copy‐edited or typeset. Technical support issues arising from supporting information (other than missing files) should be addressed to the authors.

Supplementary
