# Supplementary material for: Mapping the Binding Interface of PET Tracer Molecules and Alzheimer Disease Aβ Fibrils by Using MAS Solid‐State NMR Spectroscopy
Source: Chembiochem. 2020 May 19;21(17):2495–502. doi: 10.1002/cbic.202000143 (PMC7496087; doi:10.1002/cbic.202000143)
Supplement: Supplementary file 1 — Supplementary [file CBIC-21-2495-s001.pdf]

ChemBioChem

Supporting Information

Mapping the Binding Interface of PET Tracer Molecules and Alzheimer Disease Aβ Fibrils by Using MAS Solid-State NMR Spectroscopy

Zheng Niu, Riddhiman Sarkar, Michaela Aichler, Hans-Jürgen Wester,
Behrooz Hooshyar Yousefi+,* and Bernd Reif+*© 2020 The Authors. Published by Wiley-VCH Verlag GmbH & Co. KGaA. This is an open access article under the terms of the Creative Commons Attribution License, which permits use, distribution and reproduction in any medium, provided the original work is properly cited.This article is part of a Special Collection on the occasion of Horst Kessler's 80th birthday. To view the complete collection, visit our

Supporting Information

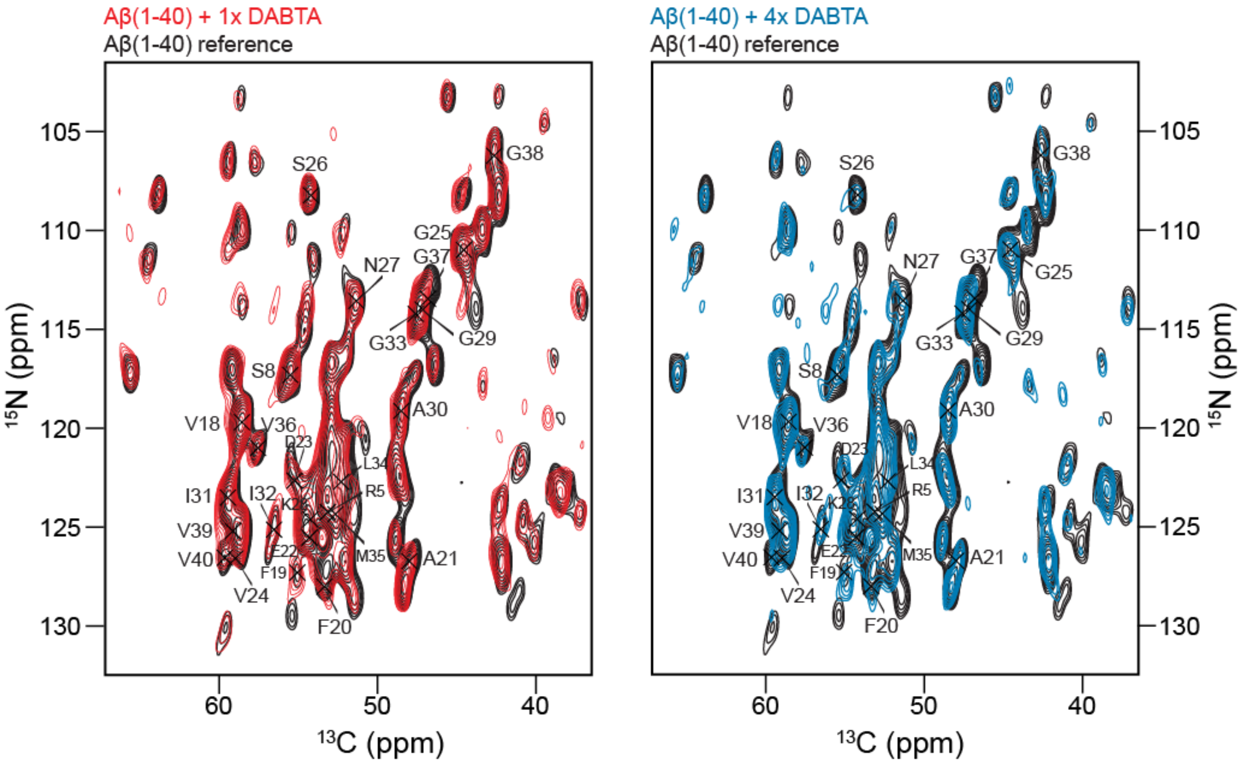

Supporting figure 1. Superposition of 2D NCACX spectra recorded for Aβ(1-40) reference fibrils (black) and Aβ(1-40) fibrils titrated with an equimolar amount of DABTA (red) and with a 4-fold molar excess of DABTA (right, blue). The experiments were carried out using a Bruker Avance III 750 MHz spectrometer. The MAS rotation frequency has been adjusted to 10 kHz. The PDSD mixing time was set to 50 ms. The effective sample temperature was 10 °C.

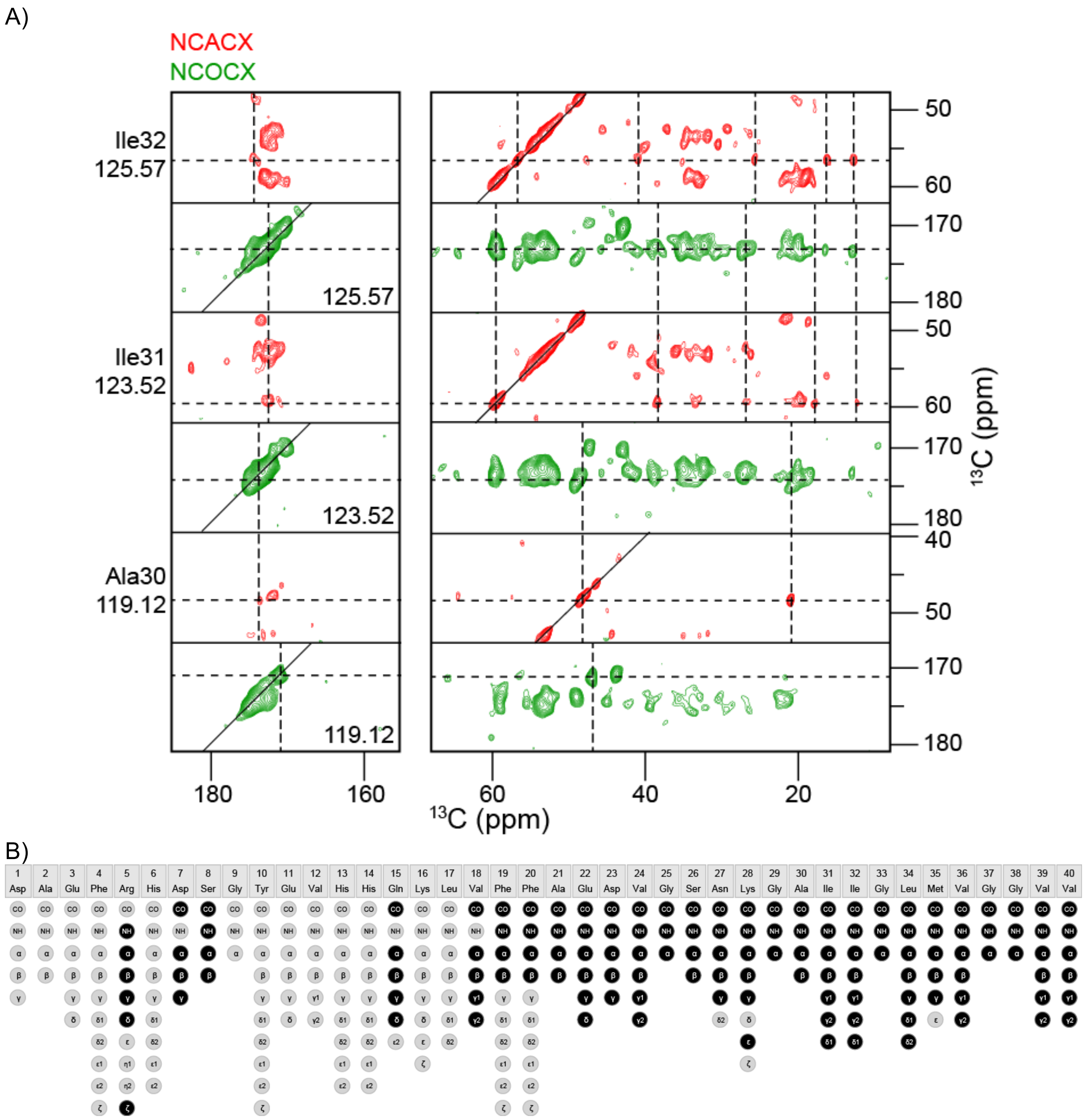

Supporting Figure 2. Chemical shift assignments of protonated Aβ(1-40) fibrils. A) Strip plots extracted from 3D NCACX and 3D NCOCX experiments showing connectivities between G29 and I32. The ^{15}N chemical shift (in ppm) of the respective amide is indicated below the residue name on the left in black. B) Schematic representation of assigned atoms in protonated Aβ(1-40) fibrils using 3D NCACX and 3D NCOCX experiments.

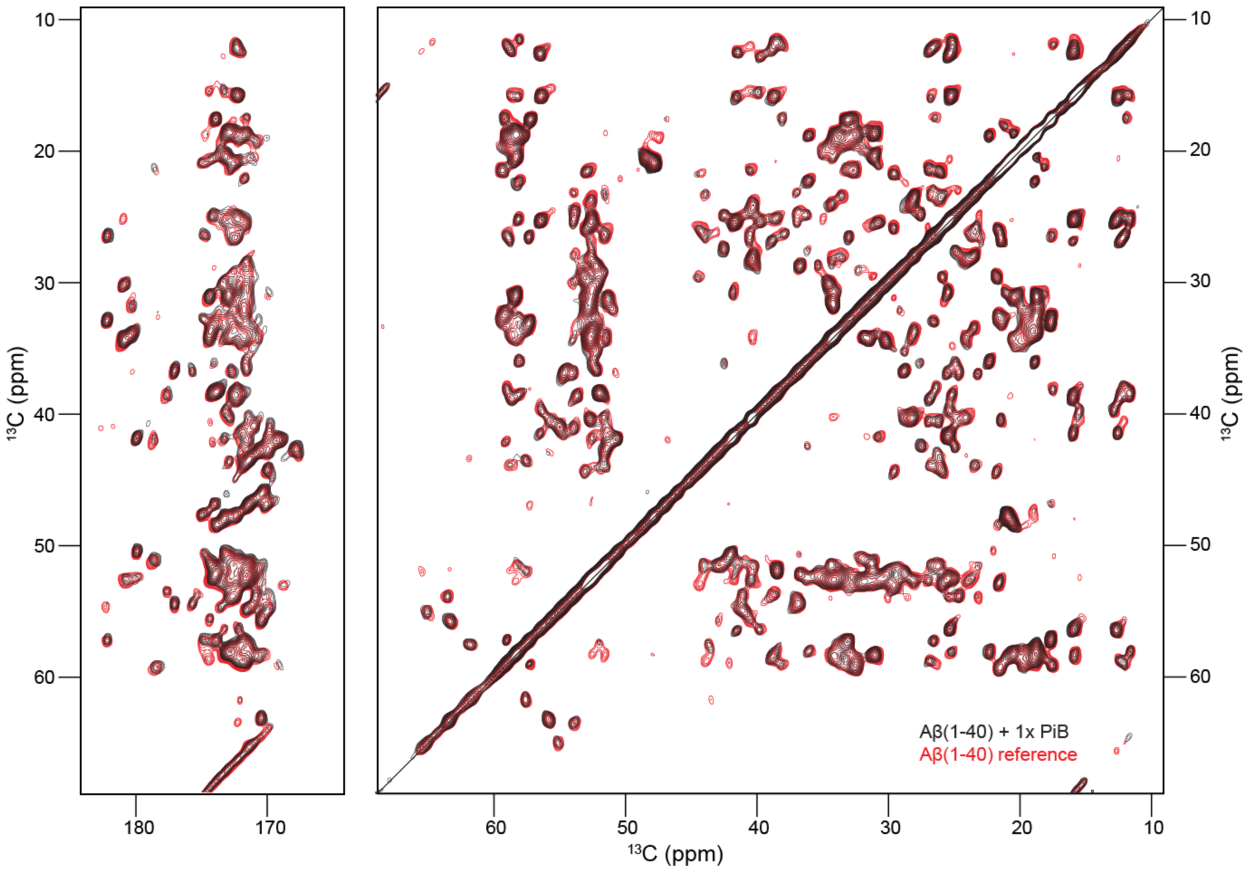

Supporting Figure 3. Superposition of 2D ^{13}C,^{13}C PDSD spectra of ^{13}C,^{15}N-labeled Aβ(1-40) reference fibrils (black) and Aβ(1-40) fibrils in the presence of an equimolar amount of PiB (red). Aβ(1-40) fibrils were produced without seeding. As a consequence, two sets of resonances are observed in the spectra. For none of the two polymorphs, chemical shift changes can be observed upon addition of PiB. The experiments were acquired using a Bruker Avance III operating at a proton Larmor frequency of 750 MHz spectrometer. The MAS rotation frequency was adjusted to 10 kHz. The PDSD mixing time was set to 50 ms.

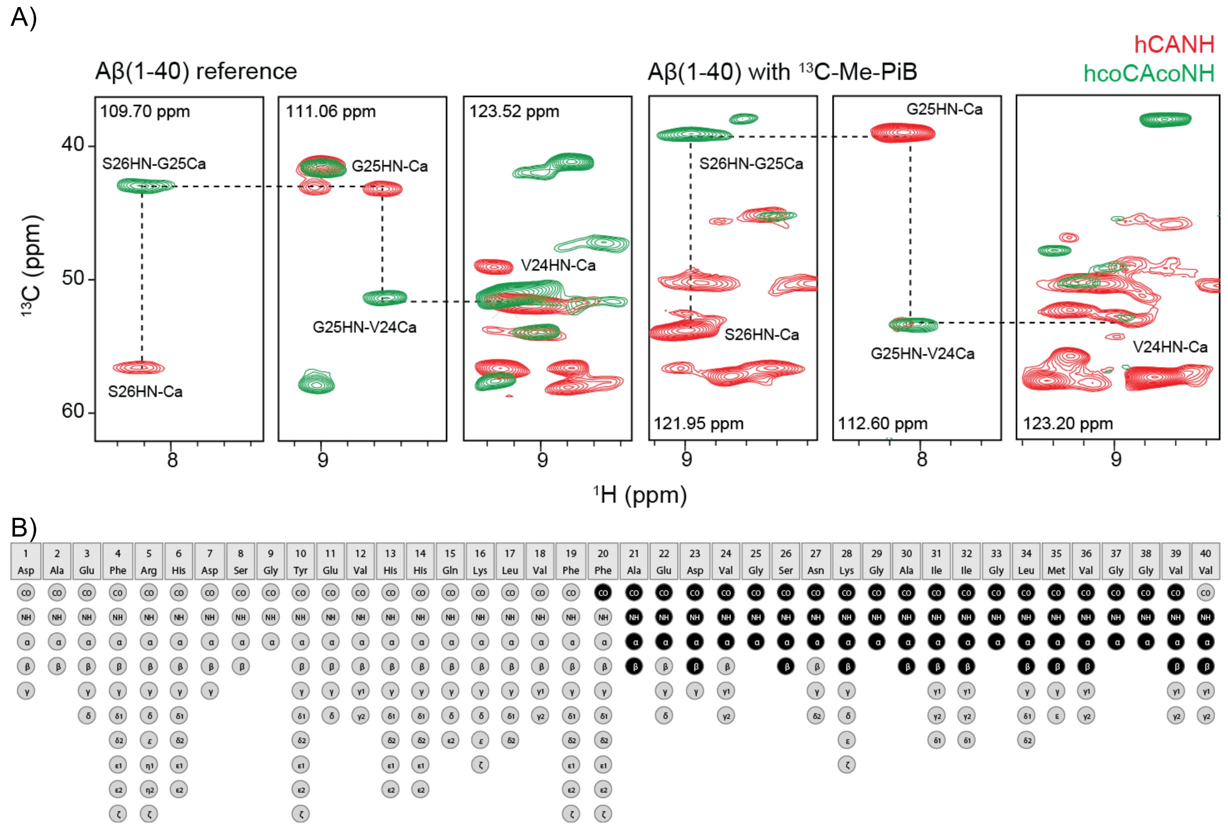

Supporting figure 4. Chemical shift assignments of deuterated Aβ(1-40) fibrils. A) Strip plots from 3D hCANH (red) and hcoCAcoNH (green) experiments for Aβ(1-40) reference fibrils (left) and for Aβ(1-40) fibrils in the presence of an equimolar amount of ^{13}C-Me-PiB. B) Assigned atoms in ^{2}H,^{13}C,^{15}N Aβ(1-40) fibrils. Assignments were obtained employing 3D hCANH, hCONH, hcaCBcaNH and hcoCAcoNH experiments.[1]

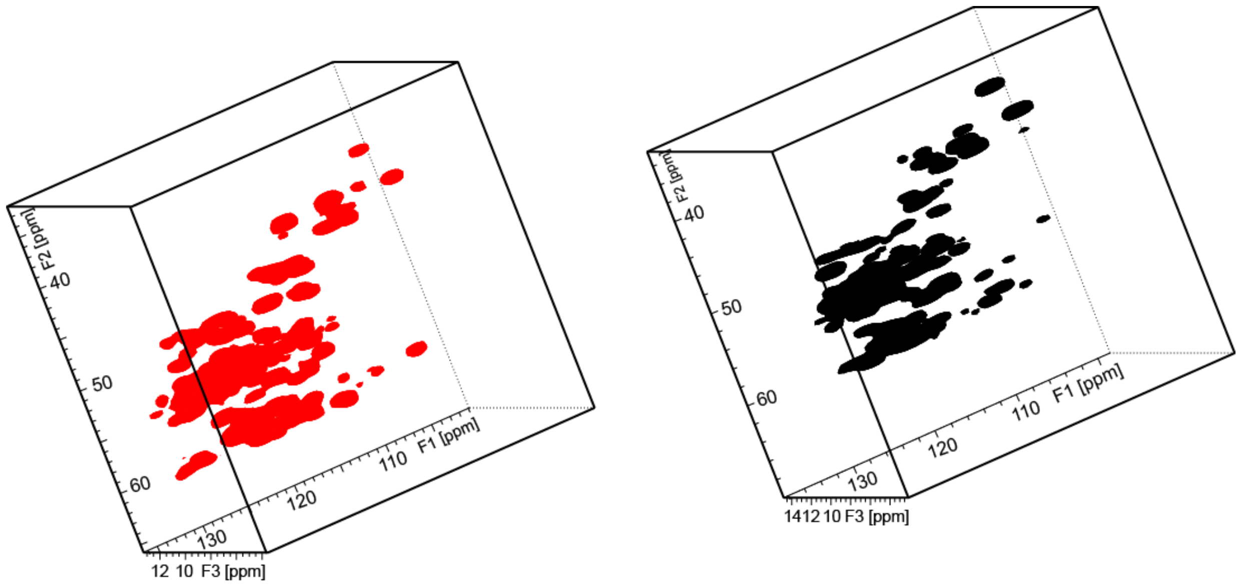

Supporting figure 5. 3D cube of hCANH experiments of Aβ(1-40) reference fibrils (left) and Aβ(1-40) fibrils in the presence of equimolar PiB (right).

Table S1. Chemical shift assignments for protonated Aβ(1-40) fibrils.

aa	N	Nζ	CO	Cα	Cβ	Cγ	Cδ	Cε	Cζ
R5	124.24	-	-	53.14	31.76	26.10	42.08	-	158.13
D7	-	-	174.49	51.87	42.00	179.10	-	-	-
S8	117.31	-	172.03	55.50	65.61	-	-	-	-
Q15	-	-	170.13	53.09	31.94	34.84	180.76	-	-
V18	119.74	-	172.38	58.81	34.46	19.20<br>20.85	-	-	-
F19	127.31	-	170.64	55.10	42.19	-	-	-	
F20	128.01	-	171.53	53.35	40.90	-	-	-	-
A21	126.72	-	173.78	47.97	19.37	-	-	-	-
E22	125.53	-	173.07	54.29	30.41	34.73	181.33	-	-
D23	122.65	-	173.54	55.24	37.89	182.71	-	-	-
V24	126.58	-	173.15	59.00	31.38	18.71<br>21.39	-	-	-
G25	111.00	-	170.44	44.72	-	-	-	-	-
S26	108.27	-	172.59	54.16	64.05	-	-	-	-
N27	113.63	-	172.63	51.21	37.18	175.10	-	-	-
K28	124.61	32.85	173.46	54.26	28.60	24.71	-	40.78	-
G29	114.00	-	171.63	47.20	-	-	-	-	-
A30	119.12	-	173.72	48.50	21.08	-	-	-	-
I31	123.52	-	172.79	59.46	38.42	26.71<br>17.87	12.45	-	-
I32	125.07	-	174.70	56.52	40.98	25.69<br>16.16	12.45	-	-
G33	114.22	-	170.51	47.57	-	-	-	-	-
L34	122.70	-	171.71	52.30	44.21	27.02	23.89	-	-
M35	124.35	-	172.13	52.67	37.16	29.16	-	-	-
V36	121.07	-	174.28	57.65	33.48	17.98	-	-	-
G37	113.46	-	171.11	46.81	-	-	-	-	-
G38	106.22	-	169.84	42.63	-	-	-	-	-
V39	125.20	-	172.82	59.17	33.20	20.00	-	-	-
V40	126.60	-	180.03	59.76	32.89	19.19<br>21.91	-	-	-

Table S2. Chemical shift assignments for deuterated Aβ(1-40) reference fibrils, and Aβ(1-40) fibrils in presence of ^{13}C-Me-PiB.

aa	H^N	N	Cα	Cβ	CO
A21	8.50<br>8.47	121.96<br>121.81	47.39<br>47.38	18.74<br>-	170.02<br>-
E22	8.30<br>-	119.17<br>-	52.02<br>-	-<br>-	171.96<br>-
D23	9.86<br>-	126.64<br>-	51.63<br>-	35.16<br>-	173.22<br>-
V24	9.27<br>-	123.63<br>-	51.47<br>54.58	-<br>-	171.85<br>171.10
G25	8.52<br>8.11	111.13<br>112.43	43.15<br>42.11	-<br>-	173.00<br>173.45
S26	8.21<br>9.08	109.70<br>118.53	56.76<br>54.59	58.07<br>-	170.56<br>171.80
N27	9.01<br>8.21	118.49<br>115.37	51.94<br>55.13	-<br>-	170.49<br>172.85
K28	8.83<br>9.10	125.63<br>128.74	53.12<br>52.65	30.69<br>-	172.44<br>171.61
G29	8.73<br>8.65	107.06<br>106.48	38.26<br>38.17	-<br>-	172.75<br>171.44
A30	7.38<br>7.33	123.91<br>123.25	49.67<br>49.68	20.48<br>-	172.48<br>171.67
I31	9.46<br>9.39	121.95<br>121.47	58.44<br>58.33	36.09<br>-	171.29<br>-
I32	9.18<br>9.11	125.20<br>125.58	58.21<br>56.53	36.58<br>-	171.79<br>172.76
G33	8.07<br>8.73	112.71<br>116.61	42.42<br>42.44	-<br>-	177.20<br>166.93
L34	8.58<br>8.61	128.95<br>128.57	50.72<br>50.73	43.41<br>-	171.91<br>171.25
M35	9.45<br>9.30	125.90<br>125.59	50.73<br>50.75	32.84<br>-	172.19<br>171.86
V36	8.57<br>8.56	127.19<br>126.97	58.05<br>58.09	29.73<br>-	175.48<br>173.73
G37	9.02<br>8.93	111.34<br>110.17	43.12<br>43.29	-<br>-	174.63<br>169.28
G38	7.88<br>7.81	106.05<br>105.20	41.22<br>41.14	-<br>-	175.43<br>167.79
V39	8.76<br>8.69	123.19<br>122.77	58.21<br>58.07	31.62<br>-	174.04<br>-
V40	9.29<br>-	132.34<br>-	58.40<br>-	-<br>-	-<br>-

References

[1] E. Barbet-Massin, A. J. Pell, J. S. Retel, L. B. Andreas, K. Jaudzems, W. T. Franks, A. J. Nieuwkoop, M. Hiller, V. Higman, P. Guerry, A. Bertarello, M. J. Knight, M. Felletti, T. Le Marchand, S. Kotelovica, I. Akopjana, K. Tars, M. Stoppini, V. Bellotti, M. Bolognesi, S. Ricagno, J. J. Chou, R. G. Griffin, H. Oschkinat, A. Lesage, L. Emsley, T. Herrmann, G. Pintacuda, J. Am. Chem. Soc. 2014, 136, 12489-12497.
